# Mammalian and Invertebrate Models as Complementary Tools for Gaining Mechanistic Insight on Muscle Responses to Spaceflight

**DOI:** 10.3390/ijms22179470

**Published:** 2021-08-31

**Authors:** Thomas Cahill, Henry Cope, Joseph J. Bass, Eliah G. Overbey, Rachel Gilbert, Willian Abraham da Silveira, Amber M. Paul, Tejaswini Mishra, Raúl Herranz, Sigrid S. Reinsch, Sylvain V. Costes, Gary Hardiman, Nathaniel J. Szewczyk, Candice G. T. Tahimic

**Affiliations:** 1 School of Biological Sciences & Institute for Global Food Security, Queens University Belfast, Belfast BT9 5DL, UK; tcahill01@qub.ac.uk (T.C.); willian.dasilveira@staffs.ac.uk (W.A.d.S.); g.hardiman@qub.ac.uk (G.H.); 2 Nottingham Biomedical Research Centre (BRC), School of Computer Science, University of Nottingham, Nottingham NG7 2QL, UK; henry.cope@nottingham.ac.uk; 3 MRC-Versus Arthritis Centre for Musculoskeletal Ageing Research and National Institute for Health Research (NIHR), Nottingham Biomedical Research Centre (BRC), University of Nottingham, Nottingham NG7 2QL, UK; joseph.bass2@nottingham.ac.uk (J.J.B.); szewczyk@ohio.edu (N.J.S.); 4 Department of Genome Sciences, University of Washington, Seattle, WA 98195, USA; eliah@uw.edu; 5 NASA Ames Research Center, Space Biosciences Division, Moffett Field, CA 94035, USA; rachelrgilbert11@gmail.com (R.G.); amber.paul@erau.edu (A.M.P.); sigrid.reinsch@nasa.gov (S.S.R.); sylvain.v.costes@nasa.gov (S.V.C.); 6 Universities Space Research Association, Columbia, MD 21046, USA; 7 Department of Biological Sciences, School of Life Sciences and Education, Staffordshire University, Stoke-on-Trent ST4 2DF, UK; 8 Department of Human Factors and Behavioral Neurobiology, Embry-Riddle Aeronautical University, Daytona Beach, FL 32114, USA; 9 Blue Marble Space Institute of Science, Seattle, WA 98104, USA; 10 Department of Genetics, Stanford University School of Medicine, Palo Alto, CA 94305, USA; tejaswini.mishra@stanford.edu; 11 Centro de Investigaciones Biológicas Margarita Salas–CSIC, Ramiro de Maeztu 9, 28040 Madrid, Spain; rherranz@cib.csic.es; 12 Department of Medicine, Medical University of South Carolina, Charleston, SC 29425, USA; 13 Department of Biomedical Sciences, Heritage College of Osteopathic Medicine, Ohio University, Athens, OH 45701, USA; 14 Department of Biology, University of North Florida, Jacksonville, FL 32224, USA

**Keywords:** spaceflight, muscle, transcriptomics, bedrest, hindlimb unloading, microgravity, animal models, cross-species comparison

## Abstract

Bioinformatics approaches have proven useful in understanding biological responses to spaceflight. Spaceflight experiments remain resource intensive and rare. One outstanding issue is how to maximize scientific output from a limited number of omics datasets from traditional animal models including nematodes, fruitfly, and rodents. The utility of omics data from invertebrate models in anticipating mammalian responses to spaceflight has not been fully explored. Hence, we performed comparative analyses of transcriptomes of soleus and extensor digitorum longus (EDL) in mice that underwent 37 days of spaceflight. Results indicate shared stress responses and altered circadian rhythm. EDL showed more robust growth signals and *Pde2a* downregulation, possibly underlying its resistance to atrophy versus soleus. Spaceflight and hindlimb unloading mice shared differential regulation of proliferation, circadian, and neuronal signaling. Shared gene regulation in muscles of humans on bedrest and space flown rodents suggest targets for mitigating muscle atrophy in space and on Earth. Spaceflight responses of *C. elegans* were more similar to EDL. Discrete life stages of *D. melanogaster* have distinct utility in anticipating EDL and soleus responses. In summary, spaceflight leads to shared and discrete molecular responses between muscle types and invertebrate models may augment mechanistic knowledge gained from rodent spaceflight and ground-based studies.

## 1. Introduction

Exposure to the unique set of spaceflight environmental conditions, including microgravity, cosmic radiation, isolation, and confinement, has been shown to induce significant physiological changes in all studied species to date [1,2,3]. These physiological responses, such as skeletal muscle atrophy, can negatively impact the health of astronauts and may also compromise mission success if left unabated [4]. The precise molecular mechanisms underlying physiological deconditioning in spaceflight have yet to be fully described, and advancement of this mechanistic understanding is essential for the development of molecularly rationalized countermeasures [5]. Such countermeasures can help maintain astronaut health during future spaceflight missions, including long duration voyages to Mars in which current countermeasures are likely to prove insufficient [6].

Studies leveraging data from omics disciplines, including transcriptomics, allow for a detailed study of the complex interactions between the molecular landscape of biological systems and spaceflight environmental conditions. Despite the scientific potential of omics datasets captured in spaceflight, only a relatively small number have been collected, mainly due to the resource-intensive nature of spaceflight experiments. NASA GeneLab was established to maximize the use of the limited number of spaceflight omics datasets. The datasets made publicly available via the GeneLab repository represent a variety of omics data types and a range of model organisms, including microbes, plants, cell cultures, vertebrates, and invertebrates [7,8]. Additionally, datasets from ground-based studies that are relevant to the spaceflight environment can be accessed via public repositories, including GeneLab and NCBI Gene Expression Omnibus (GEO) [9]. Although physiological and other functional data is required to establish clear relationships between omics changes and physiological changes, independent analysis of omics datasets is still useful for hypothesis generation, which can be used to inform future research directions. There remain numerous opportunities to use publicly available omics datasets to elucidate knowledge gaps in the field of space biology.

One such knowledge gap that currently requires further investigation is that of shared and discrete transcriptomic responses across biological tissue types, such as different muscles. Addressing this knowledge gap can prove useful for designing countermeasures that can target multiple tissues, or for providing evidence for the need to employ multiple countermeasures to provide comprehensive protection to a variety of tissues. Aside from its role in locomotion and posture, skeletal muscle plays other vital roles in whole-body health. Skeletal muscle physically shields organs and helps maintain temperature homeostasis. Additionally, as the body’s largest store of amino acids, skeletal muscle engages in crosstalk with other organs, to synthesize organ-specific proteins [10]. When the body is threatened by starvation or disease, skeletal muscle is broken down to provide other organs with energy [11].

To date, antigravity muscles in the limbs have been the main focus for studying the skeletal muscle response to spaceflight. These muscles are key to maintaining posture under 1G conditions, and are thus highly susceptible to atrophy when unloaded [12]. Certain antigravity muscles in the lower limb, such as the soleus and gastrocnemius, are also important for circulation, as upon contraction they pump venous blood back to the heart, preventing pooling and stagnation [13].

Alongside reduced muscle mass, strength, and endurance, it has been found that the mechanical unloading associated with microgravity induces a shift from type I slow twitch fibers to type II fast twitch fibers [14,15]. Previous spaceflight and ground studies in rodents and humans have also observed that antigravity muscles consisting of mainly type I (slow-twitch) fibers, which are designed for slow and sustained contraction, such as the soleus tend to be more susceptible to atrophy compared to antigravity muscles with more type II (fast-twitch) fibers [16]. Recently, Choi and colleagues reported findings from NASA’s Rodent Research-1 (RR-1) mission, in which mice were flown onboard the International Space Station (ISS) and exposed to microgravity for 37 days. They found that the weight of the soleus in space flown mice was reduced by ~19% compared to ground control mice [17]. In addition, they reported that the gastrocnemius, tibialis anterior (TA), extensor digitorum longus (EDL), and quadriceps did not show significant atrophy compared to ground control mice [17]. While significant atrophy of the soleus is consistent with findings from similar studies, the finding of non-significant atrophy in the gastrocnemius is contrary to previous space flown mouse studies [18,19,20].

Similarly, previous transcriptomic analyses of RR-1 data have reported that the soleus showed the greatest number of significant differentially expressed genes (DEGs) [21,22] compared to other muscle tissues. However, all muscle tissues did show significant gene expression changes, and hierarchical clustering of these changes indicated that the muscle tissues can be divided into two distinct groups. Group 1 consisted of plantar flexor muscles, soleus, and gastrocnemius, while group 2 consisted of dorsiflexors EDL and TA and knee extensors, quadriceps. In addition, microgravity responsive changes in immune-related pathway regulation appeared to be more prevalent in group 1 muscles compared to group 2 [21]. A different analysis of RR-1 data highlighted that changes in the expression of clock genes were fairly uniform across the muscle tissues, and yet distinct compared to other tissues such as the liver [22]. Taken together, these findings demonstrate that while certain muscles may not undergo significant atrophy in spaceflight, they still undergo molecular changes that may require intervention. Additionally, these findings support the hypothesis that in certain cases, the response to spaceflight is not identical between muscle tissues, due to their distinct morphologies and locomotive functions [16]. Further investigation is required to establish precise differences and similarities between the molecular responses of different muscle tissues. Hence in this study, we compared transcriptomic data from the fast-twitch enriched EDL, and the representative slow-twitch muscle soleus [23], collected during the RR-1 mission. We made use of two analysis approaches, Gene Set Enrichment Analysis (GSEA) and overrepresentation analysis (ORA) to determine the impact of the analysis approach on outcomes and to better anticipate the array of biological responses to spaceflight. Subsequently, to better understand the analogous nature of ground-based studies, transcriptomic data from the RR-1 quadriceps was compared to the vastus lateralis from a human bed rest study [24], and transcriptomic data from the gastrocnemius from a different space flown mouse study was compared to matching HU samples from that same study [25].

In addition to rodents, invertebrates, including *C. elegans* and *D. melanogaster*, have been established as important model organisms for understanding biological responses to the space environment [26]. While it is possible to isolate muscles in ground laboratories via dissection when the sample size of fresh animals is sufficient [27], whole organism or composite structures such as the head are typically extracted for analysis in spaceflight experiments due to logistical challenges of dissecting samples in orbit. However, insights related to muscle can still be uncovered. For example, reproducible downregulation of muscle-related genes has been observed across the “International *C. elegans* Experiment FIRST” (ICE-FIRST) and “*C. elegans* RNA Interference in Space Experiment” (CERISE) microarray experiments [28,29]. Leandro and colleagues performed a comparative analysis of space flown *C. elegans* from the ICE-FIRST experiment and space flown *D. melanogaster* from the GENE experiment [3,30]. Three analysis approaches were tested on the small subset of orthologous genes between the two organisms. Only six genes were found to have a common transcriptomic response in both experiments, with these genes all downregulated and associated with metabolic and neuromuscular signaling [3,31].

Terrestrial studies have set a strong precedent for using invertebrate models to develop tools [13,32,33] and study conserved pathways in mammalian biological processes such as aging [34,35,36,37,38]. However, the utility of omics data from space flown invertebrate studies, in anticipating aspects of mammalian responses to spaceflight, has yet to be fully investigated. With large, low cost cohorts of genetic mutants, interventional invertebrate studies are useful for establishing correlational relationships between molecular pathways and spaceflight phenotypes, with functional measures needed for causation [29]. We reason that if certain mechanisms are shared between mammals and invertebrate models, invertebrate datasets can potentially augment the limited quantity of vertebrate spaceflight omics datasets. This in turn may prove useful in anticipating mammalian responses to spaceflight. To test these concepts, we compared the abovementioned RR-1 EDL and soleus muscle datasets with transcriptomic datasets of *C. elegans* and *D. melanogaster* that were flown in the ICE-FIRST experiment and STS-121 mission, respectively. We found that spaceflight results in discrete transcriptomic signatures between representative slow and fast twitch muscles (soleus and EDL respectively), consistent with their opposing roles in flexion and differential sensitivities to unloading. In addition, transcriptomic analysis revealed that invertebrate models share select aspects of mammalian responses to spaceflight. The life stage of invertebrate models appears to impact the degree by which they recapitulate the responses of mammalian tissues to spaceflight.

## 2. Results

### 2.1. Datasets Used in This Study

Table 1 shows the transcriptomic datasets used in this study. These datasets included RNA-seq and microarray datasets. Model organisms represented are mouse, fruitfly, and nematode. A bedrest study was also selected for analysis (Refer to Methods section for further details on criteria).

### 2.2. Comparative Analysis Reveals Key Differences in Space Flown Slow and Fast Twitch Muscle

**Figure 1:**
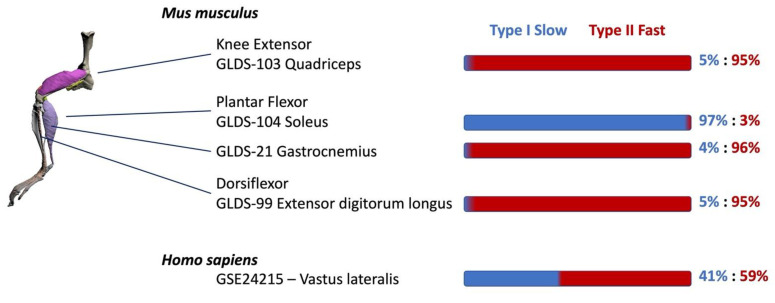
Anatomical positions of the various mouse and human muscles used in this study, grouped by function. The right panel shows the percentage of slow and fast twitch fibers for each muscle.

We first characterized the effects of spaceflight on fast and slow twitch muscle types (EDL and soleus, respectively) from the RR-1 Mission (Figure 1). Differential expression analysis (DEA) of EDL and soleus from space flown mice compared with ground controls along with ORA and GSEA are found in Supplementary Tables S1–S6, Networks S1 and S2 and Figures S1 and S2, respectively. Genes with the largest changes to their expression can provide insight on important biological processes that underlie spaceflight responses. Included in the top 10 upregulated genes in the EDL was the long non-coding RNA (*Lncbate10*) that has been shown to protect *Pgc1α* from repression [44], as well as *Mettl21e* which inhibits proteasomes [45]. We also observed upregulation of the cell cycle-related gene *Cdk1* and *Mmp12*, which play a role in ECM remodeling. The top downregulated genes included *Pde2a*, a phosphodiesterase (PDE), which controls degradation of cAMP and cGMP; the downregulation of *Pde2a* suggests increased cAMP/cGMP signaling. Downregulated as well was *Lep*, involved in energy homeostasis, and *Pck1*, a regulator of gluconeogenesis. Additionally, we found downregulation of *Stum*, which functions in mechanotransduction. Enriched results for the EDL show a response to radiation and reactive oxygen species (ROS) and the induction of apoptosis. In addition, there was enrichment of inflammatory pathways including *Stat* and *Nf-κb*, a response to wound healing and immune-related gene sets (GO:0045087, GO:0034097) involving the production of cytokines TGFβ, IL4, IL10, and type 1 interferon. Enrichment of genes related to glucocorticoid (GC) signaling, a stress response pathway, was also observed. We found overrepresentation of gene sets involved in the circadian rhythm (GO:0048511, GO:0007623) as reported previously [22,46,47,48], including downregulation of *Dbp*, a key circadian rhythm gene involved in inducing the transcription of other clock genes such as *mPer1* [49]. GSEA results also show enrichment of carbohydrate metabolism, consistent with previous studies reporting a greater reliance on glucose and a shift away from lipid metabolism [50]. In addition, we found downregulation of lipid metabolism (GO:0006631) based on ORA of downregulated genes *Lep*, *Lpl*, and *Pparγ* [51,52,53]. Analysis also revealed enrichment of lipid transport and sequestration in the EDL, consistent with previously reported lipid accumulation in atrophied muscles [50]. ORA of upregulated DEGs in the EDL show proliferation-related gene sets (GO:0008283, GO:0000278) suggesting an increase in proliferation. Conversely, GSEA revealed enrichment of mitogenic signaling pathways (MAPK), which correspond to the upregulation of genes involved in cell cycle progression (*Cdk1*). GSEA also revealed regulation of proliferation and migration of vascular endothelial cells and angiogenesis (Supplementary Network 1, Supplementary Figure S1), suggesting spaceflight-induced changes to muscle vasculature. Results also show the enrichment of muscle cell development, and differentiation as well as upregulation of *Fos*, *Mettl21e* and *Prnd*, genes involved in differentiation and the upregulation of *Pax3*, a stem cell marker expressed in activated satellite cells.

**Figure 2:**
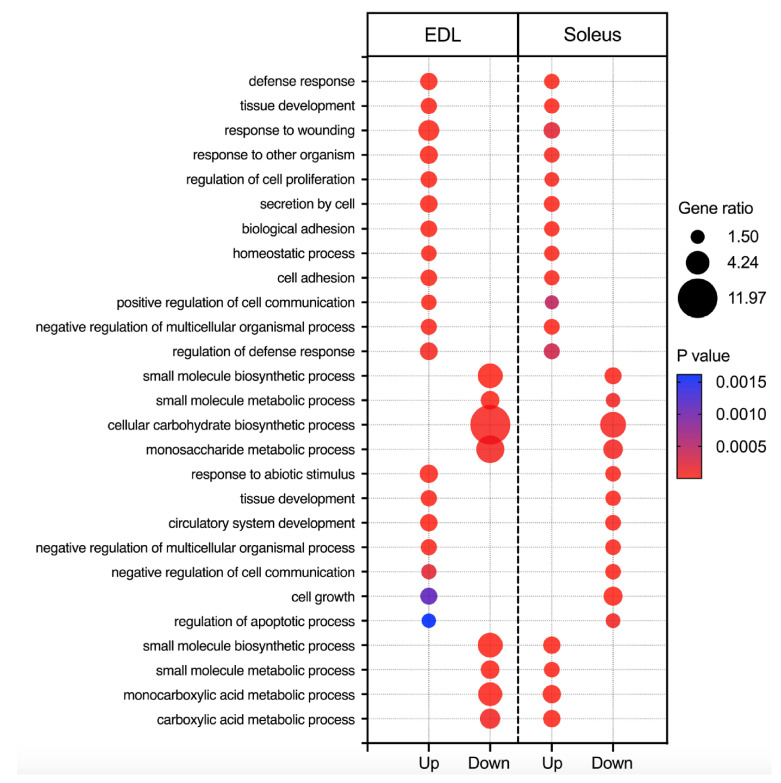
Shared upregulated and downregulated biological processes in soleus and EDL in response to spaceflight. The resulting gene ratio and *p* values from Webgestalt analysis of DEGs are shown.

In the soleus, the top upregulated genes consist of keratins, known to be important structural proteins in muscle as well as *Mettle21e*, involved in ECM remodeling, also found to be upregulated in the EDL. *Dhrs9*, involved in vitamin A biosynthesis, was downregulated, consistent with the observation of a reduction in retinol in lipid droplets in the liver of space flown mice [54]. Analyses also revealed downregulation of *Gcat*, which plays a role in threonine metabolism. Similarly, we found downregulation of *C9*, which functions in the innate immune response. Enriched results for the soleus suggest stress signaling with the enrichment of a response to GCs (GO:0051384) and the downregulation of *Ciart*, a circadian gene with a role in the transcriptional repression of *Clock* and *Bmal1* [55]. There were fewer immune related results in the soleus compared to the EDL (sole enrichment of interferon gamma and downregulation of *Il8* in the soleus). These results may suggest that the EDL has a more robust immune response than soleus, consistent with findings from a previous spaceflight study [56]. Further, GSEA indicated negative regulation of the unfolded protein response (UPR), suggesting downregulation of endoplasmic reticulum (ER) stress response in the soleus. Metabolic changes also differ in comparison to the EDL, with results indicating an increase in protein metabolism (GO:0006082, GO:0019752, GO:0072330) using upregulated genes in ORA. This is accompanied by negative correlation of ribosome biogenesis and negative nucleotide biosynthesis with spaceflight, all consistent with a decrease in muscle mass. While mixed results were reported for the regulation of lipid metabolism, the results suggest a decrease in glucose metabolism (GO:0005975). We also found enrichment of growth related gene sets such as upregulation of growth hormones including *Igf1* and its receptor, *Igf1r*. However, findings also included negative cell growth (GO:0016049) and proliferation (GO:0008283). The presence of positive and negative growth pathways may suggest remodeling of the muscles as they respond to the need to replenish and remove old or damaged cells in response to spaceflight. Similarly, results using up- and downregulated genes in ORA also indicate both positive and negative developmental cues including the enrichment of negative tissue development (GO:0009888) and cell differentiation (GO:0045595). A cross comparison of the biological processes shared between the EDL and soleus is shown in Figure 2, and shared DEGs are presented in Supplementary Table S7.

### 2.3. Molecular Signatures of *C. elegans* in Comparison with Fast and Slow Twitch Muscles during Spaceflight

#### 2.3.1. Shared Increase in Proliferation between Space Flown *C. Elegans* and Mouse Fast Twitch EDL

**Figure 3:**
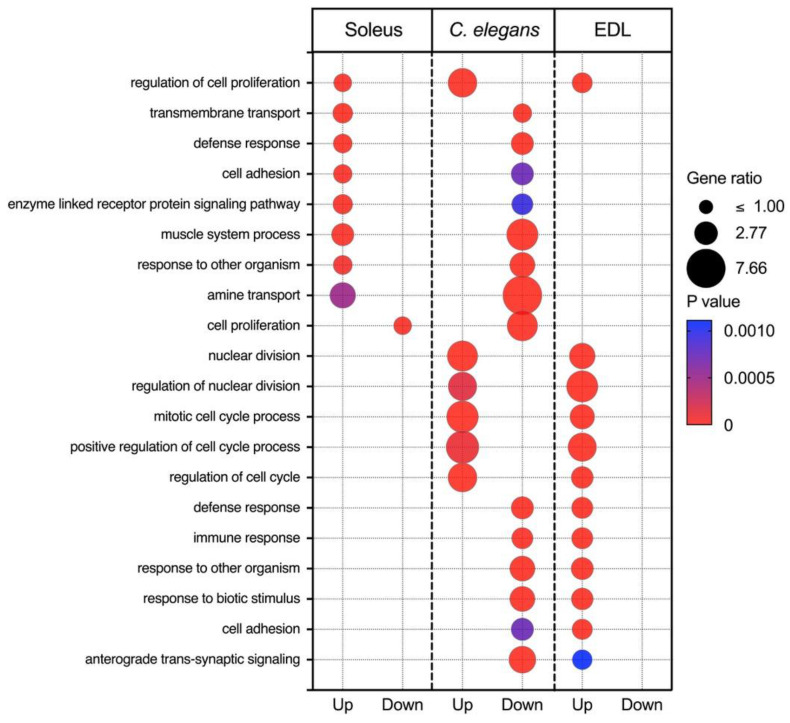
Shared upregulated and downregulated biological processes in *C. elegans* compared with soleus and the EDL in response to spaceflight. The resulting gene ratio and P values from Webgestalt analysis of DEGs are shown.

We next asked whether invertebrate models can be used to gain insight into mammalian responses to spaceflight. To address this question, we performed ORA on space flown *C. elegans* and utilized Ensembl orthology [57] to perform a cross-species comparison with mouse muscles to assess whether the transcriptional responses to spaceflight found in mammalian muscle are also observed in nematodes. DEGs and ORA can be found in Supplementary Tables S8 and S9. Differential expression analysis of space flown *C. elegans* revealed 106 DEGs (FDR < 0.05). Initial inspection of the enriched biological processes of *C. elegans* revealed that most upregulated biological processes (BP) are related to cell division (GO:0007049, GO:0051301) and development (GO:0032502), processes akin to the phenotype defined in the EDL. We also found upregulation of genes related to cytoskeleton organization (GO:0000226, GO:0007010), chitin metabolism (GO:0006030), and downregulation of ECM organization (GO:0030198). A cross-comparison between *C. elegans* and EDL enriched biological processes, shown in Figure 3, also indicates the shared overrepresentation of proliferation-related gene sets (GO:0008283, GO:0000278, GO:0022402). Gene sets related to the immune response (GO:0006955), synaptic signaling (GO:0098916) and the ECM (GO:0030198) were upregulated in the EDL but downregulated in *C. elegans.* A comparison of *C. elegans* and EDL DEGs revealed seven shared genes. These include an oncogene with a role in cell polarity (*Cab39*) [58] and a gene involved in base excision repair (*Ung*) [59]. An early development gene *Zcchc24* [60] was downregulated in both datasets. In addition, we found downregulation of *Rab20*, a gene involved in cell trafficking in the Golgi apparatus [61], consistent with downregulation of cell-cell signaling (GO:0007267). Genes upregulated in *C. elegans* but downregulated in the EDL have roles in uracil methylation (*Trmt44*), calcium-mediated synaptic transmission (*Syt12*), and thrombin degradation (*Thbd*).

#### 2.3.2. Comparison between *C. elegans* and Mouse Slow Twitch Muscle, Soleus

Similar to findings from comparisons with EDL, we also found shared biological processes between *C. elegans* and soleus (Figure 3), namely downregulation of cell proliferation. However, other processes were regulated in opposite directions. Specifically, transmembrane transport (GO:0055085), defense response (GO:0006952, GO:0098542), and cell adhesion (GO:0007155) were downregulated in C. elegans, yet upregulated in the soleus. We found 16 shared DEGs in *C. elegans* and soleus (Table 2). Upregulated in both *C. elegans* and soleus are mitochondrial genes involved in mtDNA repair (Mpv17), branched chain amino acid metabolism (*Ppm1k*) [62], and the DNA helicase gene *Mcm2*. Shared downregulated genes include Thbd, involved in thrombin degradation as well as Rab20, previously described. Genes that were upregulated in *C. elegans* but downregulated in the soleus include those involved in DNA repair (*Nsmce1*) [63], and the unfolded protein response (*Shq1*), suggesting increased damage response in C. elegans. Genes that are downregulated in *C. elegans* but upregulated in the soleus are involved in muscle excitability (*Ric3*) [64] and synaptic signaling (*Syt12*, *Ric3*, *Htr7*) [64,65,66] which may impact muscle contraction if globally upregulated in neurons. Downregulated in *C. elegans* were *Dpp4*, a gene encoding a multi-functional transmembrane protein involved in glucose uptake [67], and *Plce1* involved in growth and differentiation [68]. *Pde4b*, which codes for an enzyme that degrades cAMP to AMP, was downregulated, suggesting increased cAMP signaling in *C. elegans*, which is more akin to the EDL response.

### 2.4. Cross-Comparison between Space Flown D. melanogaster and Mouse Muscles

#### 2.4.1. Shared Stress Response in EDL and Larval *D. melanogaster*

We also determined whether space flown *D. melanogaster* share any similarities with mammalian muscle responses spaceflight. To determine this, we individually compared EDL with larvae and adult *D. melanogaster.* We reasoned that doing such may provide insight into any life-stage dependencies in our findings. DEA of space flown *D. melanogaster* larvae revealed 439 orthologous mouse genes (Supplementary Table S10), which were subjected to ORA (Supplementary Table S11). Genes were compared with mouse EDL, revealing 18 shared DEGs shown in Table 3. Shared upregulated genes include the circadian rhythm gene *Noct*, consistent with previous observations in space flown *D. melanogaster* [69]. Stress-induced molecular chaperones (*Hsp90aa1*) were also upregulated as well as genes involved in the biosynthesis of carnitine and fatty acid transport *(Tmlhe*). Shared downregulated genes include *Mettl26,* a methyltransferase. Genes with opposing patterns of regulation reveal key differences in metabolism including *D2hgdh*, upregulated in *D. melanogaster*, involved in D2-HG metabolism in the mitochondria, and *Cth*, involved in cysteine biosynthesis. Genes that were upregulated in the EDL but downregulated in *D. melanogaster* suggest differential regulation of glycosylation (*Gmppb*), the cytoskeleton (*Tubb4b*) and ECM organization (*Hspg2*, *Prcp*), blood pressure (*Prcp)*, and protein ubiquitination *(Plaa*).

#### 2.4.2. Shared Responses of Larval *D. melanogaster* and Soleus from Space Flown Mice

A comparison of larval *D. melanogaster* DEGs revealed 61 shared genes with the soleus (Supplementary Table S12). ORA of the 38 upregulated orthologs (Supplementary Table S13) suggested a shared increase in protein metabolism (GO:0006520, GO:0000096, GO:0006534). Notably, both datasets include the downregulation of *Pdia6*, which promotes proliferation [70,71] and a mitochondrial gene, *Gtpbp3*, for which studies involving its knockdown reported reduced ATP generation, increased ROS, and apoptosis [72]. The molecular chaperones *Hspa5* and *Hspb6* were also downregulated, suggesting a reduction of UPR signaling and consistent with increased protein metabolism, features observed in the soleus. *Scarb1* was downregulated in both soleus and *D. melanogaster* larvae. SCARB1 has been shown to play an important role in muscle regeneration [73]. Similarly, *Crym* was downregulated, knockout of which in mice leads to hypertrophy of fast glycolytic fibers [74]. *Pnf4*, important for actin polymerization [75] was also downregulated in both soleus and *D. melanogaster* larvae.

#### 2.4.3. Comparison of Space Flown Adult *D. melanogaster* and Fast Twitch Mammalian Muscles

We next compared adult *D. melanogaster* to the mouse muscles. Spaceflight led to 459 DEGs (FDR<0.05) in adult *D. melanogaster* (Supplementary Table S14). Of these, we found 101 that are orthologous to mouse genes, which were used for ORA (Supplementary Table S15). Four of the DEGs were found to be shared with the mouse EDL dataset. Genes that showed upregulation of expression in the two datasets were found to be involved in chromatin scaffolding (*Ppp1r10)* and replication (*Mcm6*). *Ppm1l*, downregulated in both datasets, is a negative regulator of stress and inflammatory cytokines. Hence, its downregulation in the EDL is consistent with an increased immune response [76]. Also dysregulated was *Hspg2,* which encodes an ECM protein.

#### 2.4.4. Comparison of Space Flown Adult *D. melanogaster* with Mouse Soleus

A comparison of adult *D. melanogaster* DEGs with soleus DEGs (FDR < 0.1) revealed 12 shared genes shown in Table 4. Genes that are upregulated in both datasets include a myosin protein ligase (*Mylip*) involved in muscle atrophy, previously reported to be induced by glucocorticoids [77]. Also upregulated were genes encoding a helicase protein *Mcm2* involved in replication and the pro-survival gene, *Ppm11*, which negatively regulates SAPK-mediated apoptosis. However, *Jarid2* was also upregulated in both, which represses pro-cell cycle genes [78]. Additionally, *Cbs* which takes part in the transulfuration pathways in cysteine production, is also upregulated. Genes that are downregulated in both datasets encode a nucleoporin (*Nup37*), a polymerase (*Polr2e*) and *Dnajb4*, the suppression of which has been linked to decreased growth [79]. Genes upregulated in *D. melanogaster* but downregulated in mouse soleus include the helicase subunit (*Mcm6*) and genes involved in cysteine production (*Gnmt*), calcium signaling (*Inpp5a*), and limb development (*Fjx1*).

### 2.5. Comparison between Ground-Based Unloading Models and Muscles from Space Flown Mice

#### 2.5.1. Comparison between Gastrocnemius from Hindlimb Unloaded and Space Flown Mice

To gain insight on shared transcriptomic signatures of ground-based unloading models and space flown mice, a transcriptomic analysis was conducted on RNA of gastrocnemius from space flown mice and a time and age-matched HU study (GLDS-21). Analyses revealed 75 DEGs (*p* < 0.05) in the gastrocnemius from hindlimb unloading (HU) mice (Supplementary Table S16) and 115 DEGs (*p* < 0.05) in the gastrocnemius from space flown mice (Supplementary Table S17). The top upregulated genes in the HU model have roles in UPR (*Hspa1b*, *Hspa1a*, *Atsf3*), and fatty acid biosynthesis and metabolism (*Fasn*, *Ppargc1a*). Proto-oncogenes *Fos* and *Jun* were also upregulated in HU mice. In contrast, a previous study reported upregulation of *Fos* and *Jun* in human muscle after exercise [80]. Additionally, the top 10 most downregulated genes include mitochondrial genes such as *Idh2*, which takes part in the electron transport chain, *Bdh1* involved in ketone metabolism, and *Ldhb*, which catalyzes the conversion of pyruvate to lactate. ORA performed on DEGs from gastrocnemius of HU mice (Supplementary Table S18) revealed differential regulation of stress responses (GO:0033554, GO:0009628), as well as muscle tissue proliferation (GO:0048659), differentiation (GO:0035914) and development (GO:0007519).

Conversely, the top upregulated genes in the gastrocnemius from space flown mice include *Mt1* and *Mt2*, genes that have antioxidant activity and glucocorticoid response elements [81]. We also found upregulation of a number of tumor suppressor genes such as *Btg2*, *Cebpd*, *Gadd45g*, *Cdkn1a* and *Tp53inp1* [82,83,84,85,86], suggesting reduced growth and proliferation. *Cidec*, which codes for a protein that promotes lipid droplet formation in adipocytes [87], was also upregulated. ORA of upregulated DEGs in spaceflight gastrocnemius (Supplementary Table S18) suggests a stress response as shown by differential regulation of GC signaling (GO:0071385). ORA also revealed upregulation of glucose metabolism (GO:0032868) in the gastrocnemius from space flown mice, suggesting alterations in energy homeostasis.

A comparison of DEGs in HU and spaceflight gastrocnemius revealed five shared genes with similar patterns of regulation. DEGs upregulated in both datasets suggest that HU and spaceflight both lead to altered circadian rhythm (*Nfil3*), regulation of proliferation (*Btg2*) and changes to endothelial adhesion (*Cyr61*), and scaffolding of acetylcholine receptors at the neuromuscular junction (*Musk*), which may potentially alter muscle function. Additionally, *Bdh1*, a gene involved in metabolism of ketone bodies, is downregulated in both datasets. This is consistent with a previous study showing an increase in ketone bodies such as 3-hydroxybutyrate in liver of mice flown on STS-135 [54].

#### 2.5.2. Comparison of Muscles from Bed Rest Study and Space Flown Mice

Next, we investigated shared transcriptomic responses between representative mixed fiber type muscles, and vastus lateralis muscles (VL) obtained from a bedrest study (NCBI GEO GSE24215/GLDS-370) (349 DEGs, ± 1.5 FC, *p* < 0.05, Supplementary Table S19) versus quadriceps of space flown mice (887 DEGs, *p* < 0.05), Supplementary Table S20). Eight genes were shared between these datasets (Table 5). Upregulated in both datasets was *Tbc1d12* involved in increasing glucose uptake [88], whereas the shared downregulated genes included *Ptp4a3*, the downregulation of which increases expression of ECM genes [89], and *Fbxo40*, the downregulation of which has been observed in muscular dystrophy [90]. Additionally, *Ssmpx*, a gene induced by stretching of muscles, was downregulated in the human VL but upregulated in mouse quadriceps [91]. ORA of DEGs in human VL (Supplementary Table S21) revealed downregulation of nucleotide metabolism (GO:0009117, GO:0006753) and oxidation-reduction processes usually associated with oxidative phosphorylation (GO:0055114). Conversely, ORA results for mouse quadriceps (Supplementary Table S22) showed downregulation of protein metabolism (GO:0030163, GO:0044257, GO:0051603, and GO:0006511) and a response to stress (GO:0033554).

## 3. Discussion

### 3.1. Mechanisms of Muscle Atrophy Resistance

Skeletal muscles are highly adaptive to changes in mechanical forces and display a robust regenerative capacity. In response to weight-bearing or cell damage, cells can initiate pro-survival signaling pathways that increase cell viability and growth, maintain cell number homeostasis, and allow proliferation to meet functional needs. Analysis of the transcriptomic response in the EDL from space flown animals reveals enrichment and overrepresentation of gene sets involved in necrosis, wound healing, immune response, and proliferation, which altogether may suggest a compensatory proliferation in reaction to cell damage. Since mature muscles cells do not divide, the enrichment of proliferative terms may represent the recruitment of satellite cells, which then differentiate into new myofibers [92] to achieve muscle regeneration after cellular insult [93]. This hypothesis is supported by enrichment of developmental GO terms and the upregulation of genes such as *Fos* and *Pax3* known to be expressed in activated satellite cells during muscle cell development and in early muscle regeneration post trauma [94,95]. Previous studies also show tissue regeneration markers of satellite cell activation in murine quadriceps during spaceflight [96]. Spaceflight and its analogs can lead to impaired immunity [97,98,99,100,101,102]. A more robust immune response in the EDL may also indicate damage and repair events in this muscle, given the role the immune system plays in the regeneration process [103,104]. In the EDL, the upregulation of *Il15*, which codes for an immune modulating cytokine previously noted to confer hypertrophic effects. Increased *Il15* expression may contribute to regeneration and prevent excessive atrophy in response to unloading [105]. Additionally, these results also suggest changes to vasculature, which may impact nutrient availability and therefore growth. Previous work on the HU model revealed impaired vasodilation [106] and vasoconstriction [107] in feed arteries of weight bearing plantar flexors. Similarly, radiation exposure has been shown to impact development in human vessel models [108]. The involvement of the above mentioned differentially expressed genes in skeletal muscle atrophy can be tested in future spaceflight experiments.

A genetic mechanism that may contribute to the difference in sensitivity to atrophy between the EDL and soleus may involve regulation of PDEs, which hydrolyze and tightly control cAMP/cGMP. cAMP/cGMP are ubiquitous second messenger signaling molecules known to protect against atrophy, increase myofiber size, and promote conversion to faster glycolytic fibers [109]. Our results showed downregulation of phosphodiesterase genes (*Pde2a)* in the EDL suggesting increased cAMP/gAMP signaling. In contrast, the upregulation of *Pde2a*, along with five other phosphodiesterase genes (*Pde3a*, *Pde4a*, *Pde4b*, *Pde4c*, *Pde9a*) in the soleus suggests decreased cAMP/cGMP signaling. Further work is needed to define levels of cAMP signaling in different muscle types during spaceflight and whether this pathway can be exploited to prevent muscle atrophy during spaceflight. Additionally, in the EDL, a long non-coding RNA (*Lncbate10)* was upregulated, which is known to protect *Pgc1α* from repression in adipose tissues [44]. This coincides with the upregulation of *Pgc1α*, a gene whose expression is also known to protect against atrophy [110]. The expression of *Lncbate10* has been shown to be induced by high cAMP concentrations, consistent with the downregulation of the cAMP regulator, *Pde2a* [44]. Spaceflight is known to induce slow-to-fast muscle fiber conversion [111]. In addition to *Pgc1α* upregulation in the EDL, we also observed the upregulation of *Mettl21e*, both of which are known to drive hypertrophy of type II myofibers [45,112]. Additionally, we observed the downregulation of *Vegfa* in the soleus, which was not observed in the EDL. This suggests opposing regulation of a key growth signal in vasculature of the two muscle types, which influence nutrient availability and ultimately, the propensity for muscle growth.

### 3.2. Glucocorticoids and the Circadian Rhythm

The enrichment of glucocorticoid (GC) signaling suggests that spaceflight led to upregulation of the stress response. This is consistent with the reported rise in plasma and urine cortisol levels observed in certain but not all of spaceflight studies [113,114]. GCs are steroidal signaling molecules with many roles including the regulation of energy homeostasis. They can cause the breakdown of amino acids and a decrease in insulin sensitivity, which were previously observed in the soleus [115]. GCs can also induce expression of clock genes via GC response elements and may therefore have a role in perturbing the circadian rhythm and sleep cycles during spaceflight [22,116,117,118]. The disturbance of the circadian rhythm in both muscles (EDL and soleus) may also negatively impact muscle mass. For example, knockout of a key circadian gene, *Bmal* in mice led to premature aging including sarcopenia [119]. Perturbations in GC and the circadian signaling have been implicated in disorders such as metabolic syndrome, [120,121,122], sleep disturbances [123,124], immune dysregulation [100,125,126] and cataracts [127]. Hence, these signaling pathways may potentially represent therapeutic targets for the detrimental effects of spaceflight.

### 3.3. Spaceflight Alters Mechanosensing and Neuronal Signaling in Mouse Muscle

Transcriptomic analysis of muscles from space flown mice revealed alterations in signaling pathways involved in mechanotransduction and neuromuscular communication. For example, results showed downregulation of *Stum* in the EDL, which encodes a protein important for the sensing of mechanical stimuli in proprioceptive neurons [128]. This finding is consistent with the effects of unloading and reduced proprioception observed during spaceflight [128,129]. Similarly, we observed enrichment of genes involved in Rho signaling, one of the pathways that can promote stress fiber formation [130]. Contractility changes in stress fibers is thought to be one the mechanisms by which mechanical forces exerted on the extracellular matrix (ECM) can be sensed by a cell [131]. We also found differential expression of a number of genes involved in neuron excitability such as *Grin2b,* the dysregulation of which leads to decreased muscle tone [132]. In the soleus, the enrichment of gene sets involved in nervous system development and negative axon extension and the downregulation of neurogenesis (GO:0022008) suggest changes to motor neuron signaling.

### 3.4. *C. elegans* Shows Similarity to Mouse Fast Twitch Muscle Responses to Spaceflight

Rodents have been widely used to extrapolate human responses to spaceflight due to their significant genetic, physiological, and anatomical similarities. *C. elegans*, however, have been widely used to study developmental processes since at least 83% of the proteome has a human homologue and ~8000 of its proteins have matching human gene transcripts [133]. The use of *C. elegans* as a spaceflight model has advantages over rodent models such as their relatively simpler maintenance requirements and smaller body size that allows for a larger number of individuals to be flown in space. In addition, *C. elegans* allows the use of genetic and molecular tools to dissect specific pathways and can be used for the analysis of potential countermeasures. A caveat of our comparison is that the transcriptomics was performed on a population of animals of mixed ages, and that nematodes contain multiple tissues—neurons, gut, reproductive tissue, in addition to muscles. Understanding how responses in *C. elegans* mirror mammalian responses to spaceflight may allow for better extrapolation to mammalian results. While the comparison between *C. elegans* and mouse muscles revealed differences in spaceflight-induced regulation of developmental genes, there was a shared increase in proliferation-related terms with the more atrophy resistant EDL. Similarly, we posit that *C. elegans* may resist muscle atrophy through shared increased cAMP signaling as indicated by the downregulation of *Pde4b*, encoding a cAMP regulatory enzyme, variants of which were upregulated in the soleus. The more proliferative transcriptomic signature in *C. elegans* is more similar to that of the EDL, suggesting shared mechanisms of resistance and may point to a utility for the organism in anticipating responses of fast twitch muscle fibers. Additionally, while *C. elegans* have been used for uncovering mechanisms of innate immunity analogous to humans, their immune response to spaceflight was not comparable to that seen in fast or slow twitch muscle. This may be due to the lack of an adaptive immune system in *C. elegans* [134].

### 3.5. Discrete Life Stages of D. melanogaster May Have Distinct Utility for Studying Muscle Types

Establishing shared responses to spaceflight between *D. melanogaster* and mammalian muscles also may be of value in anticipating mammalian responses to spaceflight. *D. melanogaster* are desirable as flight payloads due to their relatively small experimental footprint and their ease of maintenance. A comparison between different life stages of *D. melanogaster* and mammalian fast and slow twitch muscles revealed that discrete fly life stages may have differing utility for modeling certain features of the response to spaceflight in these mammalian muscle types. For example, an increase in protein metabolism as well as the downregulation of the UPR, two key features of the soleus response, were observed in *D. melanogaster* larvae. Conversely, protein metabolism was downregulated in adult *D. melanogaster* and not observed in the fast twitch EDL. This may suggest stage-dependent differences in metabolism. Moreover, other features of mature adult *D. melanogaster* are more akin to the fast twitch muscle, including an increase in amino acid production, calcium signaling and limb morphogenesis suggesting an environment with more growth signals. With regards to metabolism, the increase in expression of genes involved in fatty acid transport in adult *D. melanogaster* is akin to that of the EDL. However, adult *D. melanogaster* also shows a shared degradation of myosin protein with slow twitch muscle EDL. Furthermore, we also found a similar stress response between space flown adult *D. melanogaster* and mouse fast twitch muscle as indicated by differential regulation of circadian rhythm and antioxidant response genes.

Spaceflight and analog studies using *D. melanogaster* may allow for greater mechanistic insight on muscle gene regulation under spaceflight conditions. To date, on-orbit sampling of invertebrate models has mostly involved whole animals due to the logistical challenges of performing microdissections of tissues. Transcriptomic signatures can vary across muscle types and such differences may not be captured when analyzing the overall transcriptome from the whole organism. For example, each muscle type in *D. melanogaster* has a discrete pattern of isoform expression of Troponin, which regulates thin filament contraction [30,135]. We found that these isoforms are also differentially expressed in both spaceflight and analog experiments, consistent with a previous report [30]. Advances in sample preservation and in situ dissections on orbit will greatly improve our ability to gain mechanistic knowledge from smaller model organisms.

### 3.6. Differential Regulation of ECM in Spaceflight across Organismal Models

Our results indicate differential regulation of ECM-related processes in *C. elegans*, *D. melanogaster*, and rodent EDL in response to spaceflight. Specifically, *C. elegans* showed downregulation of ECM organization and cell adhesion gene sets while these were upregulated in the EDL. Similarly, there was downregulation of ECM genes *Hspg2*, *Prcp* in larval *D. melanogaster*, while these genes were upregulated in the EDL. *Hspg2* encodes Perlecan, which is involved in ECM organization [135]. PRCP activates Prekallikrein, which cleaves fibronectin, a component of the ECM [136]. *Hspg2* was also differentially regulated in adult *D. melanogaster*. Comparison of vastus lateralis from bedrest and quadriceps from space flown mice also revealed a shared upregulation of *Ptp4a3*, the downregulation of which increases expression of ECM genes [89]. These results suggest a general trend of increased expression of ECM-related genes in rodents and humans compared to downregulation in smaller model organisms such as *D. melanogaster* and *C. elegans*. The ECM plays important roles in a plethora of biological processes ranging from growth, migration, structural organization, barrier formation, and the immune response [137]. It is also impacted by spaceflight or simulated microgravity, as part of the mechanotransduction pathways involving integrins and mechanically activated ion channels [138]. The differences in the direction of regulation of ECM-related genes may be due to differences in body or tissue structure (hence differing ECM composition) and the differences in the timing of sampling and duration of flight. However, differential expression of ECM-related genes in all spaceflight models and analogs tested in this study suggest that ECM remodeling is likely to be a universal response to spaceflight.

### 3.7. Transcriptomic Signatures of Spaceflight Models and Analogs Exhibit Similarities and Key Differences

Ground-based analogs such as HU have proven useful in anticipating many aspects of the musculoskeletal response to microgravity. Analysis of the transcriptome of the gastrocnemius from space flown rodents and a time-matched HU study revealed differences in transcriptomic signatures between these two models, particularly in the direction of regulation of genes involved in cell proliferation, circadian rhythm, endothelial cell adhesion, and motor neuron signaling. These changes are consistent with musculoskeletal atrophy, gait changes, sleep disturbances, and altered vascular function in humans that experienced spaceflight. However, we recognize that experimental limitations may impact comparative analysis of ground-based and spaceflight studies in rodents. Specifically, differences in animal handling and housing of space and ground-based rodent models can potentially affect the results. The use of appropriate housing controls and introduction of manipulations in controls that consider the additional handling associated with sending payloads to space (e.g., transport in ground vehicles, landing forces, light-dark cycle changes) may minimize the confounding factors when comparing findings from ground-based versus spaceflight rodent studies.

We also compared muscles from a rodent spaceflight and human bedrest study, two of the relatively resource intensive approaches to modeling human responses to spaceflight. Both have key advantages relative to ground-based rodent models. The former is expected to reflect mammalian responses to actual spaceflight and the later, actual human physiological responses to unloading. We found that representative mixed fiber type muscles in HU mice and humans on bedrest (quadriceps and VL, respectively) share similarities in differential expression of *Tbc1d12*, a gene involved in glucose uptake. In addition, *Fbxo40*, a gene that is also upregulated in a denervation model for muscle atrophy [90] is similarly responsive to both spaceflight in rodents and bedrest. Further, FBXO40 functions as a muscle-specific E3 ubiquitin ligase that is regulated by activated STAT3, thereby increasing insulin resistance in mice under 1G conditions [139]. Upregulation of both *Tbc1d12* and *Fbxo40* in bedrest and rodent spaceflight models is consistent with reported perturbations in glucose metabolism observed in humans in space [140]. These findings provide a rationale for testing whether targeting *Fbxo40* and its signaling partners will be useful in mitigating muscle atrophy and altered glucose metabolism in both spaceflight and Earth-based scenarios.

## 4. Materials and Methods

### 4.1. Datasets Used in This Study

Table 1 lists the various datasets used in this study. These datasets can be found in NASA GeneLab and are cited in these references [24,25,37,38,39,40,41]. The datasets involving mice flown on the ISS make use of the NASA Rodent Research notation for the various experimental groups. Specifically, NASA RR-1 validation mice consisted of four groups to also enable assessment of the contributions of age and cage configuration. These include Spaceflight (FLT) and Ground Control (GC) groups, which both make use of the NASA rodent habitat. The GC group was run with a 4-day delay to allow for replication of actual temperature, gas partial pressures, and humidity conditions of the FLT group. In addition, a baseline (Basal) group was euthanized on Earth one day after launch to compare changes in both the FLT and GC groups. Lastly, a Vivarium (Viv) control comprising mice maintained in standard housing was also included to determine the impact of the mouse habitat hardware in the results obtained from the FLT and GC groups. Refer to [17] for further details. NASA GeneLab generated omics data from the following comparisons: Basal vs Viv controls, GC vs Viv controls, FLT vs GC [17]. In this work, we focus on the GC and FLT groups. A number of bedrest studies focusing on muscle have been published, with a variety of assay approaches (coding and non-coding RNA), main experimental variables, and tissue collection schemes [24,141,142]. For this current analysis, we selected a transcriptomic dataset from a previous bedrest study [24] based on availability of raw expression data from coding RNA.

### 4.2. Processed RNAseq Data

Differential expression data of RNA-seq datasets (GLDS-99, 103 and 104) were downloaded from NASA GeneLab. The NASA GeneLab online database describes the standard analysis pipeline used to generate this processed data. Briefly, the percentage of rRNA in raw fastq files was assessed using HTStream SeqScreener (version 1.0.0) and then filtered using Trim Galore! (version 0.6.2). The quality of both raw and trimmed reads was evaluated with FastQC [143] (version 0.118), while MultiQC [144] (version 1.7) was used to generate MultiQC reports. Indexes for Mus musculus genome were generated with genome version mm10-GRCm38 (Mus_musculus.GRCm38.dna.toplevel.fa), and Mus_musculus.GRCm38.96.gtf using STAR [145] (version 2.7.1a). Processed reads were aligned to the *Mus musculus* reference with STAR (version 2.7.1a) and aligned reads were then quantified using RSEM [146] (version 1.3.1). Quantification data was imported to R [147] (version 3.6.0) with tximport [148] (version 1.14.0) and normalized with DESeq2 [149] (version 1.26.0). Differential expression analysis was conducted in R (version 3.6.0) using DESeq2 (version 1.26.0). All groups were compared using the Wald test and the likelihood ratio test was used to generate the F statistic p value. Gene annotations were assigned using the following Bioconductor and annotation packages: STRINGdb [150], PANTHER.db [151], and org.Mm.eg.db [152]. Differential expression analysis using DESeq2 [153] was performed on expression data from space flown subjects against ground controls to assess gene level changes in these muscles and use in downstream pathway analysis.

### 4.3. Processing of Microarray Data

For Affymetrix microarray datasets (GLDS-3 and GLDS-21), the raw expression data were downloaded from NASA GeneLab database. The data were normalized using the ‘affyNormQC.R’ r script applying the RMA algorithm through the oligo R package with default parameters. The ‘affyNormQC.R’ r script was also used to generate quality control with parameter ‘do.logtranspaceflightorm’ set to TRUE. The microarray experiments were annotated with the r script ‘annotateProbes.R’, which employed Annotation-Db class probe annotations specific to the chip used in each experiment from the Bioconductor repository. In cases where multiple probes mapped to the same gene ID, representative probes were selected with the highest mean normalized intensity across all samples. The limmaDiffExp.R r script was used to perform differential gene expression analysis on normalized expression data to perform pair-wise comparisons for all groups. For each probe set, the variance of mean signal intensities was estimated, improved by an empirical Bayes method for combining variances of probes showing similar variability, and the significance of the difference between the means was evaluated with a *t*-test to obtain *p* values. *p* values were adjusted for multiple hypothesis testing using the Benjamini and Hochberg method to control the false discovery rate [154,155]. Raw Agilent microarray data (GLDS-113 and GLDS-370) were downloaded from NASA GeneLab. Data were analyzed using Gene Spring software (Agilent Technologies, Santa Clara, CA, USA). Background correction was performed using the ‘normexp’ method (with offset = 50), and between array normalization was performed utilizing the quantile normalization method with a log2 transformation. Control probes and those without a RefSeq ID were removed, while probes mapping to the same RefSeq ID were collapsed by mean expression (leaving ~20,700 genes for analysis). All datasets used are outlined in Table 1 and anatomical locations of mammalian muscles are shown in Figure 1.

### 4.4. Pathway Analysis

First, differential expression analysis (DEA) was performed to reveal DEGs, the top 10 of which sorted by fold change values were used to pinpoint central players that orchestrate the response to spaceflight. ORA using WebGestalt [156] was then performed on DEGs to determine enriched gene ontology terms (Log2 FC > 0.32 or < -0.32, FDR adjusted *p* < 0.05). A variance stabilizing transformation was applied to the count data from datasets GLDS-99 and GLDS-104, and a Gene Set Enrichment Analysis was performed with FDR threshold of < 0.1 using Cytoscape [157]. The EnrichmentMap and Autoannotate Cytoscape plugins were used to visualize and annotate clusters of overlapping gene sets to help identify overarching enriched functional themes and aid in the interpretation of the effects of spaceflight in distinct muscle types. A comparative analysis was performed on the results from each muscle.

## 5. Conclusions

This study aimed to determine the effects of spaceflight on the transcriptome of distinct mammalian muscle types and to define shared transcriptomic signatures across a variety of model organisms. Our analyses revealed that spaceflight elicited both shared and discrete responses in representative slow and fast twitch muscle types (soleus and EDL respectively). The shared responses between these muscle types include altered expression of genes involved in GC stress responses and the circadian rhythm. These two muscle types displayed differences in transcriptomic signatures pertaining to immune function and cellular growth, with the EDL exhibiting greater degree of differential regulation of these processes based on the number of differentially expressed genes between spaceflight and ground samples. Additionally, EDL and soleus showed differences in the transcriptomic response of genes involved in ER stress mechanisms. Our findings also highlight the possible role of *Pde2a* as a key molecule that may confer the atrophy resistance seen in the EDL. We also found that the muscle specific Ubiquitin E ligase *Fbxo40* was downregulated across models for muscle atrophy (bedrest and spaceflight), suggesting a possible target for countermeasures development. These results were also used as a backdrop for the comparison to spaceflight responses of non-mammalian models. The molecular signature of *C. elegans* in response to spaceflight showed greater similarity to that of the EDL. Furthermore, the transcriptomic signature of the larval stage of *D. melanogaster* showed more similarity to the slow twitch muscle soleus. In contrast, the response of adult *D. melanogaster* was more akin to that of fast twitch muscle EDL. Muscles from rodent spaceflight and human bedrest studies indicate alterations in glucose homeostasis and circadian rhythm which are consistent with findings in humans that experienced spaceflight.

As expected for a highly resource intensive data collection endeavor, spaceflight omics datasets are rare and can have differences in experiment designs. One of the advantages of applying unbiased bioinformatics analyses of gene expression data is that patterns, differences, or similarities in the regulation of biological processes can be observed from a relatively small number of datasets. We have demonstrated that such an approach can be used to continue to gain insight on the shared responses across model organisms and also between ground and flight analogs for microgravity. However, we recognize limitations in this study, including the possibility that differing experiment designs across datasets such as duration of flight, relative life stage and gender and sex can impact the results obtained. In addition, several datasets yielded a relatively small number of differentially expressed genes, which can limit the ability to find shared molecular signatures across multiple datasets. As more spaceflight and analog datasets become available from the various model organisms, follow-up analyses can be conducted using the approach we have employed. We anticipate that doing so will reveal additional shared mechanisms across the model organisms of spaceflight. Although the use of invertebrate models such as *D. melanogaster* and *C. elegans* show promise in anticipating select spaceflight responses of specific muscles, our findings also confirm the marked differences in molecular signatures across these model organisms when dissections are not possible. Hence, the use of rodents to extrapolate human responses to spaceflight continue to have major advantages from a physiology perspective versus classic non-mammalian genetic model systems such as *D. melanogaster* and *C. elegans*. However, our findings raise the possibility of using invertebrate models as a first step toward conducting precision animal research in future deep space missions.

## Figures and Tables

**Table 1:** Datasets used in the study, including the organism’s name, duration of spaceflight or unloading, assay type, sex, strain, tissue, age, and sample size. Refer to GeneLab and GEO databases for further details. GC: ground control, FLT: spaceflight, VC: vivarium control, HU: hindlimb unloading, Gastroc: gastrocnemius, Quad: quadriceps, VL: vastus lateralis, F: female, M: male, Mx: mixed; H: hermaphrodite. Tg: Gal4-UAS transgenic line expressing two copies of eGFP from the hemolectin promoter. N: number of individuals, rep: replicate of pooled samples run for transcriptomic analysis. Refer to [24,25,37,38,39,40,41,42,43] for links to GeneLab processed data and versions used in this study.

Dataset	Organism	Duration	Vehicle	Assay	Sex	Strain	Tissue	Age/Stage	Sample Size
GLDS-104	*M. musculus*	37 d	ISS	RNAseq	F	C57BL/6J	Soleus	16 wks	N = 6 (GC); N = 6 (FLT)
GLDS-99	*M. musculus*	37 d	ISS	RNAseq	F	C57BL/6J	EDL	16 wks	N = 6 (GC); N = 6 (FLT)
GLDS-21	*M. musculus*	11 d, 19 h	STS-108	Microarray	F	C57BL/6J	Gastroc	9 wks	N = 4 (GC); N = 4 (FLT)
GLDS-21	*M. musculus*	12 d	N/A (HU)	Microarray	F	C57BL/6J	Gastroc	9 wks	N = 5 (VC); N = 5 (HU)
GLDS-103	*M. musculus*	37 d	ISS	RNAseq	F	C57BL/6J	Quad	16 wks	N = 6 (GC); N = 6 (FLT)
GLDS-370/GEO GSE24215	*H. sapiens*	10 d	N/A (Bedrest)	Microarray	M	N/A	VL	24-27 yrs	N = 10 (Longitudinal)
GLDS-3	*D. melanogaster*	12 d, 18.5 h	STS-121	Microarray	Mx	Tg	Whole organism	3rd instar larvae	N = 50/rep × 6 (GC); N = 50/rep × 6 (FLT)
GLDS-3	*D. melanogaster*	12 d, 18.5 h	STS-121	Microarray	F	Tg	Whole organism	Adults	N = 20/rep × 3 (GC); N = 20/rep × 3 (FLT)
GLDS-113	*C. elegans*	10 d	ISS	Microarray	H	N2	Whole organism	Mixed stage	N ≈ 10000/rep × 3 (GC); N ≈ 10000/rep × 3 (FLT)

**Table 2:** DEGs shared between space flown *C. elegans* and soleus muscle and their direction of regulation. Values shown are Log2 fold change (FC).

Genes	*C. elegans*	Soleus
**Both upregulated**		
*Mcm2*	0.4	0.38
*Mpv17*	0.39	0.32
*Ppm1k*	0.33	0.58
**Both downregulated**		
*Rab20*	−0.4	−0.48
*Thbd*	−0.64	−0.45
**Opposite regulation**		
*Nsmce1*	0.5	−0.41
*Tmem205*	0.39	−0.78
*Shq1*	0.36	−0.36
*Exosc3*	0.33	−0.34
*Ppp4r4*	−0.32	1.63
*Syt12*	−0.32	0.72
*Dpp4*	−0.36	0.55
*Ric3*	−0.37	0.49
*Htr7*	−0.38	0.77
*Plce1*	−0.5	0.39
*Pde4b*	−0.51	0.73

**Table 3:** DEGs shared between space flown *D. melanogaster* and EDL and their direction of regulation. Values shown are Log2 FC.

Genes	Larval *D. melanogaster*	EDL
**Both upregulated**		
*Tmlhe*	0.99	0.34
*Noct*	0.61	0.59
*Hsp90aa1*	0.51	0.53
**Both downregulated**		
*Chac1*	−0.91	−1.61
*Mettl26*	−0.43	−0.38
**Opposite regulation**		
*Amdhd2*	0.4	−0.46
*Adck5*	0.37	−0.5
*D2hgdh*	0.85	−0.32
*Cth*	0.49	−0.5
*Surf6*	0.56	−0.39
*Tubb4b*	−0.36	0.53
*Gmppb*	−0.33	0.33
*Ypel2*	−1.15	0.65
*Prcp*	−0.59	0.32
*Cotl1*	−0.7	0.41
*Plaa*	−0.36	0.32
*Timm9*	−0.42	0.42
*Hspg2*	−0.45	0.33

**Table 4:** DEGs shared between space flown adult *D. melanogaster* and soleus muscle and their direction of regulation. Values shown are Log2 FC.

Genes	Adult *D. melanogaster*	Soleus
**Both upregulated**		
*Mylip*	0.7	0.41
*Ppm1l*	0.69	0.46
*Mcm2*	0.69	0.38
*Cbs*	0.42	2.09
*Jarid2*	0.89	0.39
**Both downregulated**		
*Nup37*	−0.33	−0.39
*Polr2e*	−0.36	−0.47
*Dnajb4*	−0.33	−0.41
**Opposite regulation**		
*Gnmt*	0.72	−0.6
*Inpp5a*	0.44	−0.86
*Fjx1*	0.41	−0.54
*Mcm6*	0.37	−0.79

**Table 5:** DEGs shared between human bedrest VL and space flown mouse quadriceps and direction of expression. Values shown are Log2 FC.

Genes	Vastus Lateralis	Quadriceps
**Both upregulated**		
*Tbc1d12*	0.84	0.29
*Lonrf3*	1.09	0.7
**Both downregulated**		
*Ptp4a3*	−1.26	−0.61
*Mgst3*	−1.11	−0.41
*Fbxo40*	−1.1	−0.17
*C7orf50*	−0.89	−0.46
**Opposite regulation**		
*Smpx*	−1.09	0.30
*Hccs*	−0.84	0.30

## Data Availability

The transcriptomic datasets that support the findings of this study are publicly available in NASA Genelab and NCBI Gene Expression Omnibus. All other data associated with this study are found in the main manuscript and supplementary section.

## Supplementary Materials

The following are available online at https://www.mdpi.com/article/10.3390/ijms22179470/s1.
