# Supplementary figures and images for: Mammalian and Invertebrate Models as Complementary Tools for Gaining Mechanistic Insight on Muscle Responses to Spaceflight
Source: Int J Mol Sci. 2021 Aug 31;22(17):9470. doi: 10.3390/ijms22179470 (PMC8430797; doi:10.3390/ijms22179470)

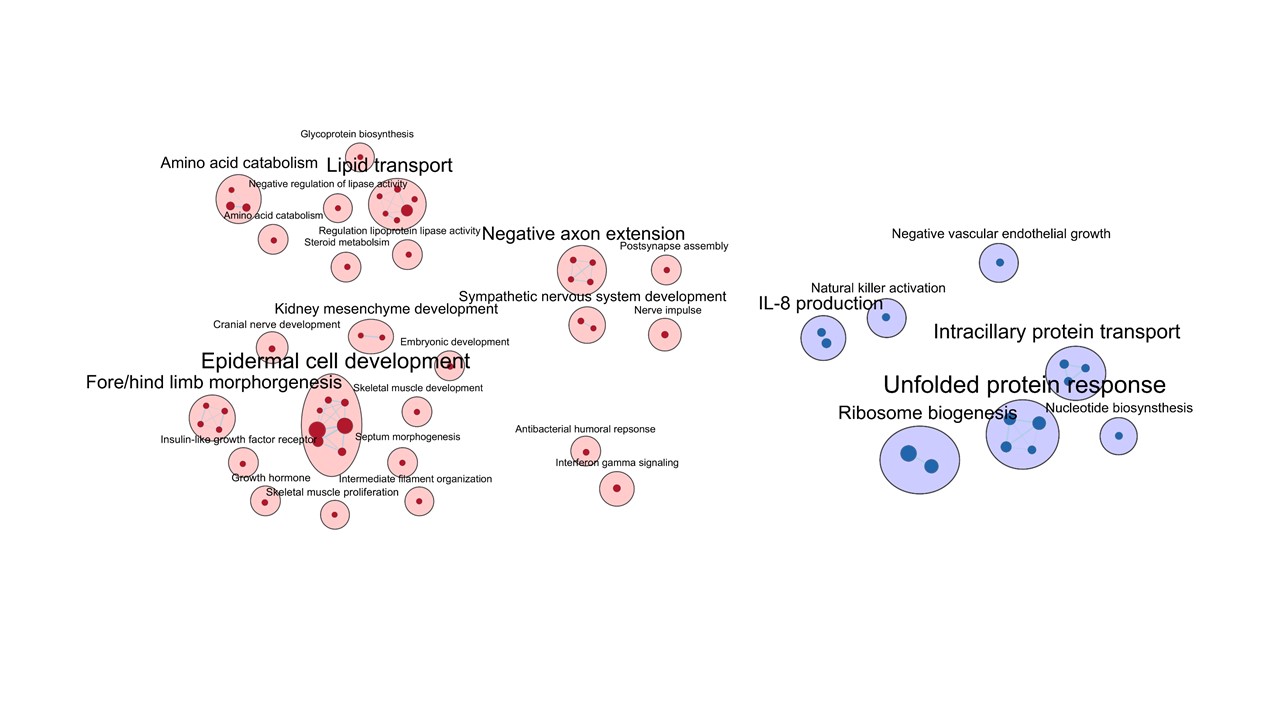

Supplement: Supplementary file 1 [file ijms-22-09470-s001.zip › Supplementary material/Figure_S2_GSEA_GLDS_104_SLS_FLT_vs_GC_GO_BP.jpg]

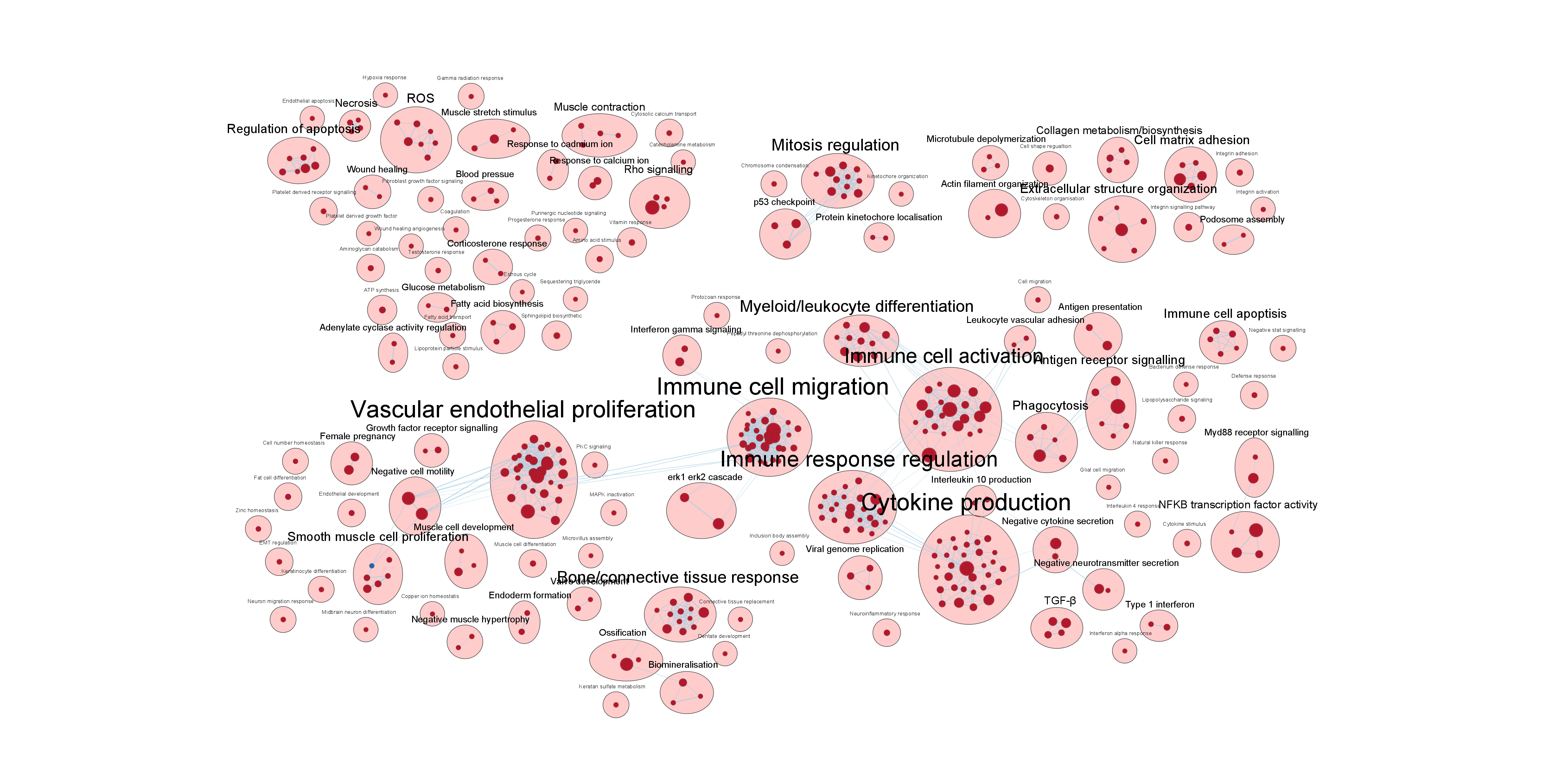

Supplement: Supplementary file 1 [file ijms-22-09470-s001.zip › Supplementary material/Figure_S1_GSEA_GLDS_99_EDL_FLT_vs_GC_GO_BP.png]
